# MicroRNA-21 Regulates PI3K/Akt/mTOR Signaling by Targeting TGFβI during Skeletal Muscle Development in Pigs

**DOI:** 10.1371/journal.pone.0119396

**Published:** 2015-05-07

**Authors:** Lijing Bai, Ruyi Liang, Yalan Yang, Xinhua Hou, Zishuai Wang, Shiyun Zhu, Chuduan Wang, Zhonglin Tang, Kui Li

**Affiliations:** 1 State Key Laboratory for Animal Nutrition, Key Laboratory of Farm Animal Genetic Resources and Germplasm Innovation of Ministry of Agriculture of China, Institute of Animal Science, Chinese Academy of Agricultural Sciences, Beijing, China; 2 Agricultural Genome Institute at Shenzhen, Chinese Academy of Agricultural Sciences, Shenzhen, China; 3 National Engineering Laboratory for Animal Breeding, Key Laboratory of Agricultural Animal Genetics and Breeding, Department of Animal Breeding and Genetics, College of Animal Sciences and Technology, China Agricultural University, Beijing, China; University of Cincinnati, College of Medicine, UNITED STATES

## Abstract

MicroRNAs (miRNAs), which are short (22–24 base pairs), non-coding RNAs, play critical roles in myogenesis. Using Solexa deep sequencing, we detected the expression levels of 229 and 209 miRNAs in swine skeletal muscle at 90 days post-coitus (E90) and 100 days postnatal (D100), respectively. A total of 138 miRNAs were up-regulated on E90, and 31 were up-regulated on D100. Of these, 9 miRNAs were selected for the validation of the small RNA libraries by quantitative RT-PCR (RT-qPCR). We found that miRNA-21 was down-regulated by 17-fold on D100 (P<0.001). Bioinformatics analysis suggested that the transforming growth factor beta-induced (TGFβI) gene was a potential target of miRNA-21. Both dual luciferase reporter assays and western blotting demonstrated that the TGFβI gene was regulated by miRNA-21. Co-expression analysis revealed that the mRNA expression levels of miRNA-21 and TGFβI were negatively correlated (r = -0.421, P = 0.026) in skeletal muscle during the 28 developmental stages. Our results revealed that more miRNAs are expressed in prenatal than in postnatal skeletal muscle. The miRNA-21 is a novel myogenic miRNA that is involved in skeletal muscle development and regulates PI3K/Akt/mTOR signaling by targeting the TGFβI gene.

## Introduction

MicroRNAs (miRNAs) are small, non-coding RNA molecules that modulate gene expression through the translational repression or the deadenylation/degradation of target mRNAs [1, 2]. Many studies have documented that miRNAs play an important roles in myogenesis. Several miRNAs have been identified as critical regulators of muscle development. In particular, the miR-1/206 and miR-133a/133b families contribute to the development of myocardial and skeletal muscles [3, 4]. In C2C12 myoblast, miR-1 suppresses the expression of HDAC and promotes myoblast differentiation [5]. miR-133 promotes C2C12 myoblast proliferation by repressing the serum response factor (SRF) [3]. The miR-206 induces C2C12 myoblast differentiation by down-regulating the DNA polymerase α subunit (polyα) [6] and connexin 43 (Cx43) [7]. In zebrafish, miR-214 positively regulates slow muscle-associated phenogenetics [8]. Our group has found that miR-148a is a novel myogenic miRNA that promotes myogenic differentiation by repressing the ROCK1 gene [9].

Transforming growth factor beta-induced (TGFβI) gene, which is otherwise known as betaig-h3, was first identified through the induction of its expression by TGF-β in human lung carcinoma cell lines [10]. As one member of the TGF-β superfamily, TGFβI has been linked to embryonic development, adult tissue homeostasis, and disease pathogenesis. In developing mouse embryos, TGFβI is expressed in most mesoderm-derived tissues and has been shown to accumulate at high levels at myotendinous junctions as well as being present on the surfaces of skeletal muscle fibers [11]. FAS1 domains of TGFβI have been reported to inhibit tumor angiogenesis and growth [12]. TGF-β superfamily signaling is initiated by the phosphorylation of the cytoplasmic signaling molecules Smad2 and Smad3, which participate in the TGF-β/activin pathway, or Smad1/5/8, which is involved in the bone morphogenetic protein (BMP) pathway. TGF-β signaling can also affect Smad-independent pathways, including the Erk, p38 MAPK, and PI3K pathways [13]. PI3K activates downstream target proteins, such as Akt and mTOR, to promote the rearrangement of the cytoskeletal elements associated with diver se cellular biological processes.

The pig is an important farm animal and an ideal model for biomedical and disease research. Recent studies have identified miRNA involvement in swine skeletal muscle development [14–16]. These miRNAs substantially affect animal development and skeletal muscle phenotypes. Our group has reported that miR-378 is a potential myogenic miRNA that regulates skeletal muscle development by targeting BMP2 and MAPK1 [17]. The miR-155 plays a role in prenatal skeletal muscle development by mediating the expression of the olfactomedin-like 3 (OLFML3) gene in pigs [18]. The miR-1 regulates skeletal muscle development by targeting CNN3 in pigs [19].

Chinese native pig breeds are significantly differ from western breeds in terms of growth rate, muscle mass and meat quality. However, no studies have examined the functions of miRNAs in the skeletal muscle development of indigenous Chinese pigs. To elucidate the molecular mechanisms underlying the characteristic muscle phenotype of Chinese native pigs, we explored Tongcheng pigs (a famous indigenous breed) as the model for this study. We profiled the miRNA transcriptome in the skeletal muscle at 90 days post coitus (E90) and 100 days postnatal (D100) and found that miR-21 was significantly (17-fold) differentially expressed. The miR-21 has been implicated in carcinogenesis, and it is up-regulated in many cancers [20–23]. However, no studies have been performed investigating the expression and function of miR-21 in pigs’ myogenesis to date. In this study, we demonstrated that miR-21 was a potential novel, myogenic miRNA. Bioinformatics prediction analysis suggested that TGFβI was a putative target of miR-21. We observed that miR-21 regulated PI3K/Akt/mTOR signaling by targeting TGFβI based on dual luciferase and western blot assays. Co-expression analysis revealed that their expressions at the mRNA level were negatively correlated in the skeletal muscle during the 28 developmental stages.

## Materials and Methods

### Tissue sample collection and RNA isolation

Skeletal muscle samples were collected from Tongcheng pigs at 90 days post coitus (dpc) and from piglets from 100 days postnatal (dpn) for small RNA sequencing. Meanwhile, we collected the longissimus dorsi at 15 prenatal developmental stages (33-, 40-, 45-, 50-, 55-, 60-, 65-, 70-, 75-, 80-, 85-, 90-, 95-, 100-, and 105- dpc) [17] and 13 postnatal stages (0-, 9-, 20-, 30-, 40-, 60-, 80-, 100-, 120-, 140-, 160-, 180- dpn and adulthood). Nine tissues, including heart, liver, spleen, lung, kidney, small intestine, ovary, uterus, and longissimus dorsi tissues, were collected from adult Tongcheng sows for spatial expression analysis. Three biological replicates of every sample were collected for each tissue and developmental stage. All animal procedures were performed according to the protocols approved by Hubei Province, PR China for the Biological Studies Animal Care and Use Committee (Permit Number:[1993]79). All surgery was performed under sodium pentobarbital anesthesia, and all efforts were made to minimize suffering. All tissues were harvested and immediately frozen in liquid nitrogen. The homogenization of the tissue samples was performed using TRIzol (Invitrogen).

### Small RNA library preparation and data analysis

RNAs were extracted from the longissimus dorsi of the fetuses (E90) and adults (D100). Then, the population of recovered small RNAs, which ranged in size from 18–30 nt, were purified, and adaptors were ligated to their 5’ and 3’ ends. Reverse transcription reactions were performed, and 90-bp DNA fragments were isolated from the agarose gel. Thereafter, the samples were delivered to the Beijing Genomics Institute for sequencing via Solexa technology.

After the initial reads were obtained from the Solexa sequencing reaction, those with low-quality tags, 5’ adaptor pollution sequences, poly(A) stretches, and sequences of less than 18 nt and those without 3’ adaptors and insertions were removed, All clean reads were compared to the Rfam database (http://www.sanger.ac.uk/software/Rfam) and GenBank non-coding RNA database (http://www.ncbi.nlm.nih.gov/) for the annotations of the rRNAs, tRNAs, scRNAs, snRNAs, and snoRNAs. Known miRNAs were identified by comparing our clean tags to mature miRNAs in the miRBase 20.0 database (http://www.mirbase.org). All the data obtained were deposited in the NCBI Sequence Read Achieve (http://www.ncbi.nlm.nih.gov/Traces/sra/) with the accession NO.SRP050975.

### RT-PCR and real-time quantitative PCR

RNA was extracted from the cells and different tissues, and the total RNA (1 μg) was reverse-transcribed into cDNA using the RevertAid First Strand cDNA Synthesis Kit (Fermentas). The differentially expressed miRNAs were validated using stem-loop RT-PCR primers as shown in S1 Table. Real-time PCR was performed using the Applied Biosystems 7500 Real-time PCR System with SYBR Premix Extaq (Takara), according to the manufacturer’s protocols. The housekeeping genes GAPDH and U6 were used as internal normalization controls for the mRNA and miRNA, respectively. The PCR mixtures contained 10 μL 2X SYBR Premix Extaq, 0.4 μL 50X ROX Reference Dye II, 0.4 μL forward and reverse primers respectively, 2 μL cDNA, and RNase-free and DNase-free H_2_O up to a final volume of 20 μL. Then, the reaction mixtures were incubated in a 96-well plate at 95°C for 10 min, followed by 40 cycles at 95°C for 5 s and 60°C for 34 s. All samples, in addition to the no-RT and no-template controls, were analyzed in triplicate. The experimental data were analyzed by the 2^−ΔΔCT^ method using the 7500 System SDS Software version 1.4.0 [17].

### Target prediction and KEGG pathway analysis

To determine the regulatory target of miR-21, TargetScan (version 6.2) was used to predict the putative targets (http://www.targetscan.org/). The predicted targets were classified according to the KEGG functional annotations using the DAVID Bioinformatics Resource (http://david.abcc.ncifcrf.gov/tools.jsp), and those potential targets involved in regulating muscle development were selected for further analysis.

### Cell culture and transfection

Pig iliac endothelial cells (PIEC; Center for Type Culture Collection, China) were cultured in Dulbecco’s modified Eagle’s medium (Gibco) supplemented with 10% FBS (Gibco), 1% penicillin/streptomycin (Gibco), and 1% Glu Max (Gibco).

The miR-21 mimics (double-stranded RNA oligonucleotides) and negative control duplexes, the miR-21 inhibitor (single-stranded RNA oligonucleotides) and inhibitor negative control duplexes were synthesized by GenePharma. Transfection was performed with the Lipofectamine 2000 reagent (Invitrogen), which was combined with 50 nM of the miRNA mimics and the negative control.

### Target validation using a luciferase reporter gene assay

The region of the TGFβI mRNA 3’ UTR flanking the miR-21 binding site was amplified from the Tongcheng pig genomic DNA using specific primers (TGFβI 3’ UTR sense, TGFβI 3’ UTR antisense, S2 Table). The PCR product was cloned into a region downstream of the Renilla luciferase ORF (Promega) using the Not I and Xho I restriction sites. The resulting plasmid was named psi-check2-TGFβI-3’UTR. Additionally, we obtained a psi-check2 luciferase reporter vector containing the mutant 3’ UTR of TGFβI and sharing a 7-bp deletion in the conserved miR-21 binding site from Shanghai Generay Biotech.

The PIEC cells were co-transfected with 200 ng psi-check2-TGFβI-3’UTR plasmid/ TGFβI-3’UTR mut and 20 pM miR-21 mimics/negative control using the Lipofectamine 2000 reagent (Invitrogen) in 24-well plates. After transfection for 48 hours, the cells were harvested. Renilla and firefly luciferase activities were measured with the Dual Luciferase Assay System (Promega).

### Western blotting

After the PIEC cells were transfected for 48 hours with miR-21 mimics/negative control and miR-21 inhibitor/inhibitor negative control, cell proteins were extracted using the T-PER Mammalian protein extraction Reagent (Pierce), and E90 and D100 longissimus dorsi proteins were isolated with T-PER Tissue Protein Extraction (Thermo) according to the manufacturer’s protocol. The extracts were stored at -80°C.

The proteins were separated by SDS-PAGE, transferred and immunoblotted with antibodies to TGFβI (Abcam), β-actin (Cell Signaling), p-Akt (Cell Signaling), Akt (Cell Signaling), p-mTOR (Cell Signaling) and mTOR (Cell Signaling). The signals got from the western blotting were quantified with the Image J program (NIH).

### Statistical analysis

Quantitative data are presented as the mean ± SD and were analyzed using Student’s test. All analyses were performed with SPSS 16.0. Statistical significance was defined at *P*<0.05.

## Results

### An overview of the small RNA transcriptome

**Fig 1:**
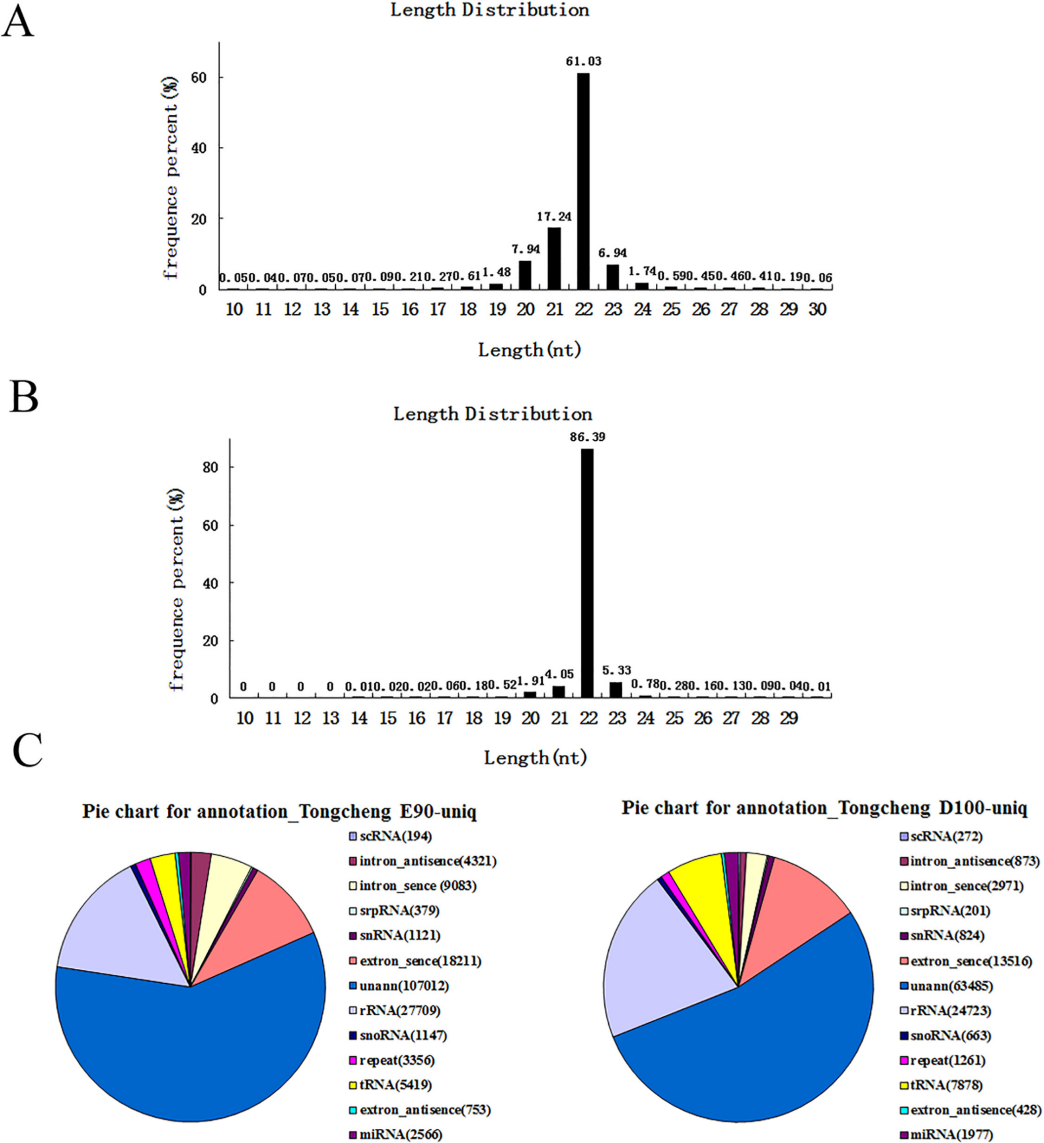
Deep-sequencing results and annotations of small RNAs from swine skeletal muscle. A. Size distribution of sequenced small RNAs from E90. B. Size distribution of sequenced small RNAs from D100. C. Annotations of sequenced small RNAs.

A total of 11,355,046 reads and 17,867,081 reads were obtained for E90 and D100, respectively. After the removal of the reads lacking the 3’ primer, those without insert tags, those with polyA or with 5’ primer contaminants, and those smaller than 18 nt, we finally obtained 10,366,627 high-quality clean reads at E90 and 17,690,485 at D100. The largest proportion of small RNAs were 22 nt in length, followed by 21–23 nt, consistent with the 21–23 nt range of the mature miRNAs (Fig 1A and 1B). To assess the efficiency of deep sequencing for the detection of the miRNAs, all sequence reads were annotated and matched to rRNAs, snoRNAs, and tRNAs (S3 Table). Based on the data from miRBase (version 20.0), Rfam (version 9.1) and the NCBI GenBank database, most of the annotated small RNAs were sus-miRNAs (ssc-miRNAs), comprising approximately 50% of the total sequence reads (Fig 1C).

### Top 15 abundant miRNAs in skeletal muscles at E90 and D100

The distribution of the top most abundant 15 miRNAs in our Solexa sequencing analysis are shown in Table 1. Of these, ssc-miR-206 had the highest reads in the E90 libraries, and ssc-miR-1 was highly expressed at D100. These two miRNAs were considered to be myomiRs, and the data herein confirmed that ssc-miR-206 and ssc-miR-1 play vital roles in muscle development. Aside from ssc-miR-206 and ssc-miR-1, ssc-miR-378 was the most abundant at E90, followed by ssc-miR-143-3p, ssc-let-7a, ssc-let-7f, ssc-let-7c, ssc-miR-30d, ssc-miR-30a-5p, ssc-miR-10b, ssc-miR-127, ssc-miR-148a, ssc-miR-126, ssc-miR-7i, and ssc-miR-21. These 15 aforementioned miRNAs accounted for 6.43% of the total miRNA reads detected in the muscle at E90, while the same reads accounted for only 4.83% at D100.

**Table 1:** The top 15 abundant miRNAs in sus skeletal muscles by deep sequencing.

miR Name	E90	D100	Tissue distribution	Targets in skeletal muscles
ssc-miR-1	65414	720939	Muscle-specific	HDAC[3],myocardin [24],cyclinD1[25]
ssc-miR-206	239878	74149	Muscle-specific	Pax7[26], DNApoly-α[6], cx43[7]
ssc-miR-378	154024	2445	Cardiac-enriched	IGF1R[27],GRB2[28]
ssc-miR-143-3p	60432	226	ubiquitous	ORP8[29],MyoD[30],SRF[31],Myocardin[31], Nkx2-5[31]
ssc-let-7a	41839	25619	ubiquitous	
ssc-let-7f	31813	25184	ubiquitous	
ssc-let-7c	31292	4422	ubiquitous	
ssc-miR-30d	10930	412	ubiquitous	
ssc-miR-30a-5p	7083	256	ubiquitous	
ssc-miR-10b	7013	123	ubiquitous	
ssc-miR-127	5762	25	ubiquitous	
ssc-miR-148a	4635	23	ubiquitous	ROCK1[9]
ssc-miR-126	2475	3	ubiquitous	Spred-1[32],VCAM-1[32], IRS-1[33]
ssc-let-7i	2341	754	ubiquitous	
ssc-miR-21	2139	126	ubiquitous	

Previous studies have reported different miRNAs involved in myogenesis. Among the top 15 miRNAs in our data, we found 6 (40%) miRNAs which have been validated by in vitro/vivo experiments about their kay roles in skeletal muscle development. Of them, ssc-miR-21 has been recognized as the most prominent miRNA implicated in carcinogenesis. However, no studies have been conduction assessing the expression and function of miRNA-21 in myogenesis. Based on the progress of miR-21 in kinds of cell lines, we speculated that miR-21 might take part in the myogenesis of porcine skeletal muscle.

### Differentially expressed miRNAs between E90 and D100

A total of 138 miRNAs were up-regulated in the skeletal muscle at E90, while 31 were up-regulated at D100 (S1 Fig). These differentially expressed miRNAs may play important roles in porcine muscle development. As presented in Table 2, one class showed 100-fold greater levels of expression at E90 compared to D100, which included ssc-miR-126, ssc-miR-143-3p, ssc-miR-127, ssc-miR-148a, ssc-miR-196b-5p, and ssc-miR-369; another class exhibited expression levels of were slightly lower than 100-fold, which included ssc-miR-542-3p, ssc-miR-99b, ssc-miR-378, ssc-miR-30a-5p, ssc-miR-10b, and ssc-miR-21. Four miRNAs (ssc-miR-29a, ssc-miR-1, ssc-miR-128, ssc-miR-320) showed obviously enhanced expression at D100 compared to E90.

**Table 2:** MiRNAs differentially expressed between E90 and D 100.

miR Name	D100/E90	E90/D100	miR Name	D100/E90	E90/D100
ssc-miR-126		875.57	ssc-miR-196a		45.66
ssc-miR-143-3p		267.2	ssc-miR-151-3p		43.05
ssc-miR-127		230.62	ssc-miR-382		39.82
ssc-miR-148a		198.54	ssc-miR-30e-3p		35.69
ssc-miR-196b-5p		160.05	ssc-miR-24		33.94
ssc-miR-369		103.44	ssc-miR-30a-5p		27.72
ssc-miR-542-3p		87.56	ssc-miR-30d		26.55
ssc-miR-99b		75.36	ssc-miR-30e-5p		17.14
ssc-miR-378		62.99	ssc-miR-21		17
ssc-miR-30a-3p		57.22	ssc-miR-29a	56.87	
ssc-miR-10b		56.96	ssc-miR-1	11.02	
ssc-miR-100		56.92	ssc-miR-128	5.13	
ssc-miR-148b		50	ssc-miR-320	2.32	

(Fold change >10.0, p<0.001 and FDR<0.001).

Highly abundant miRNAs may fulfill a more complex function in the regulation of muscle development. Compared to these miRNAs, those were less enriched and may also be important for muscle development, particularly due to their increased abundance in specific tissues or at specific developmental stages. Therefore, studies investigating novel miRNAs that are expressed at relative low levels but are nonetheless functionally important are becoming increasingly urgent. Of the miRNAs identified, we found that ssc-miR-21 was up-regulated by 17-fold in the skeletal muscle on E90. The findings of miR-21 can contribute to our current knowledge of porcine myomiRs.

### RT-qPCR validation of differentially expressed miRNA

**Fig 2:**
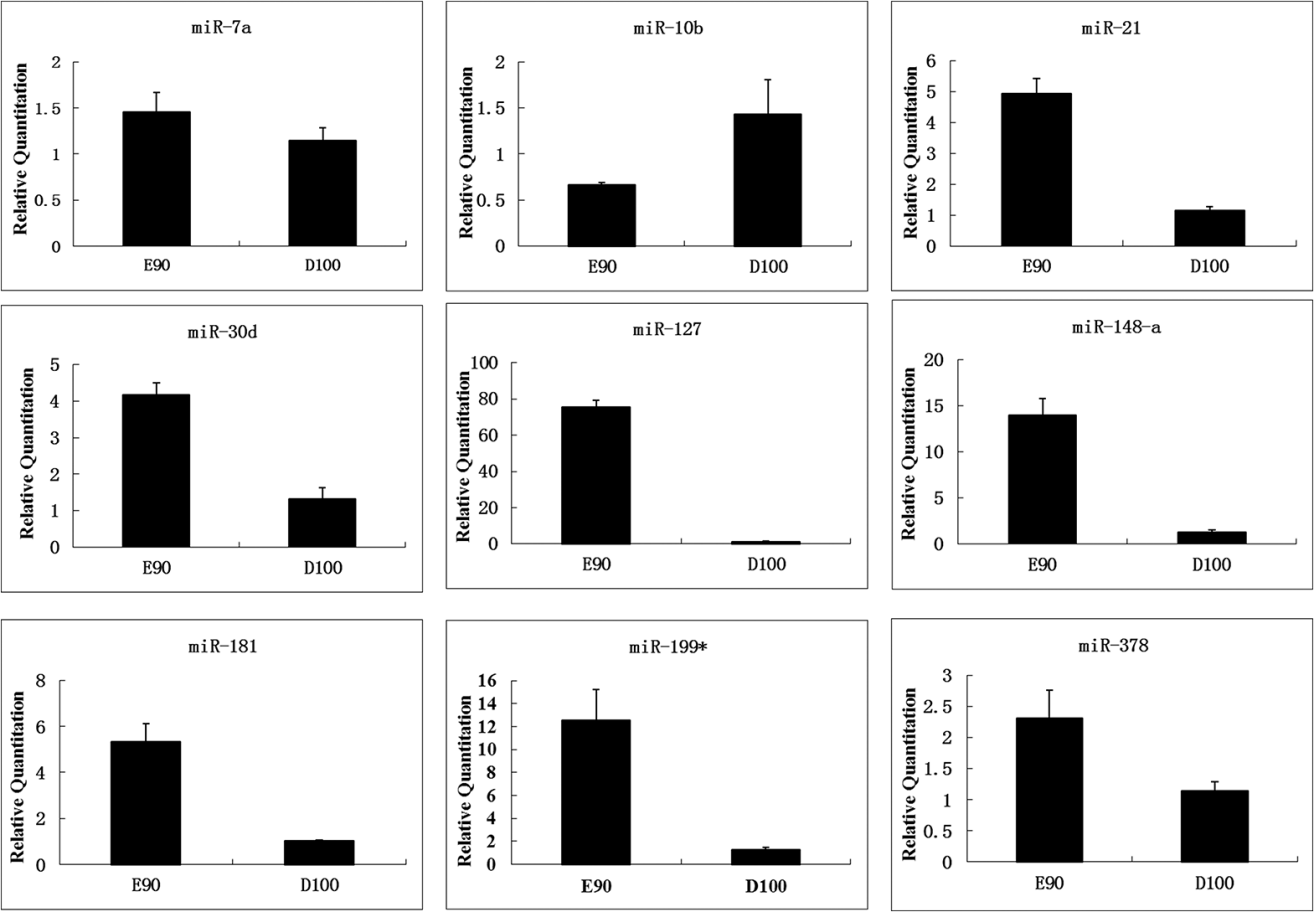
The expression of muscle-related miRNAs during the skeletal muscle development of Tongcheng pigs was detected using real-time PCR. The miRNAs obtained from the longissimus dorsi of the Tongcheng pigs between E90 and D100 were evaluated. At least 3 animals were used for each time point, and the expression of the miRNAs was normalized to that of U6.

Nine differentially expressed miRNAs (ssc-miR-7a, ssc-miR-10b, ssc-miR-21, ssc-miR-30d, ssc-miR-127, ssc-miR-148a, ssc-miR-181, ssc-miR-199*, and ssc-miR-378) were chosen for the validation of the Solexa sequencing data via RT-qPCR. The expression patterns of the miRNAs assayed by RT-qPCR were in accordance with the Solexa sequencing results with the exception of ssc-miR-10b, which showed opposite results, being expressed at higher levels in the skeletal muscle at D100 than at E90 (Fig 2). Notably, the expression levels of ssc-miR-127 and ssc-miR-148a were much higher in the skeletal muscle at E90 than at D100, indicating that they both play roles in the promotion of myogenesis. Ssc-miR-21, ssc-miR-30d, ssc-miR-181, ssc-miR-199*, and ssc-miR-378 were all expressed at higher levels at the prenatal compared with the neonatal stage, and their distinct expression patterns during muscle development clearly reflected the relationship between miRNA and myogenesis.

### Target prediction and pathway analysis for miRNA-21

**Fig 3:**
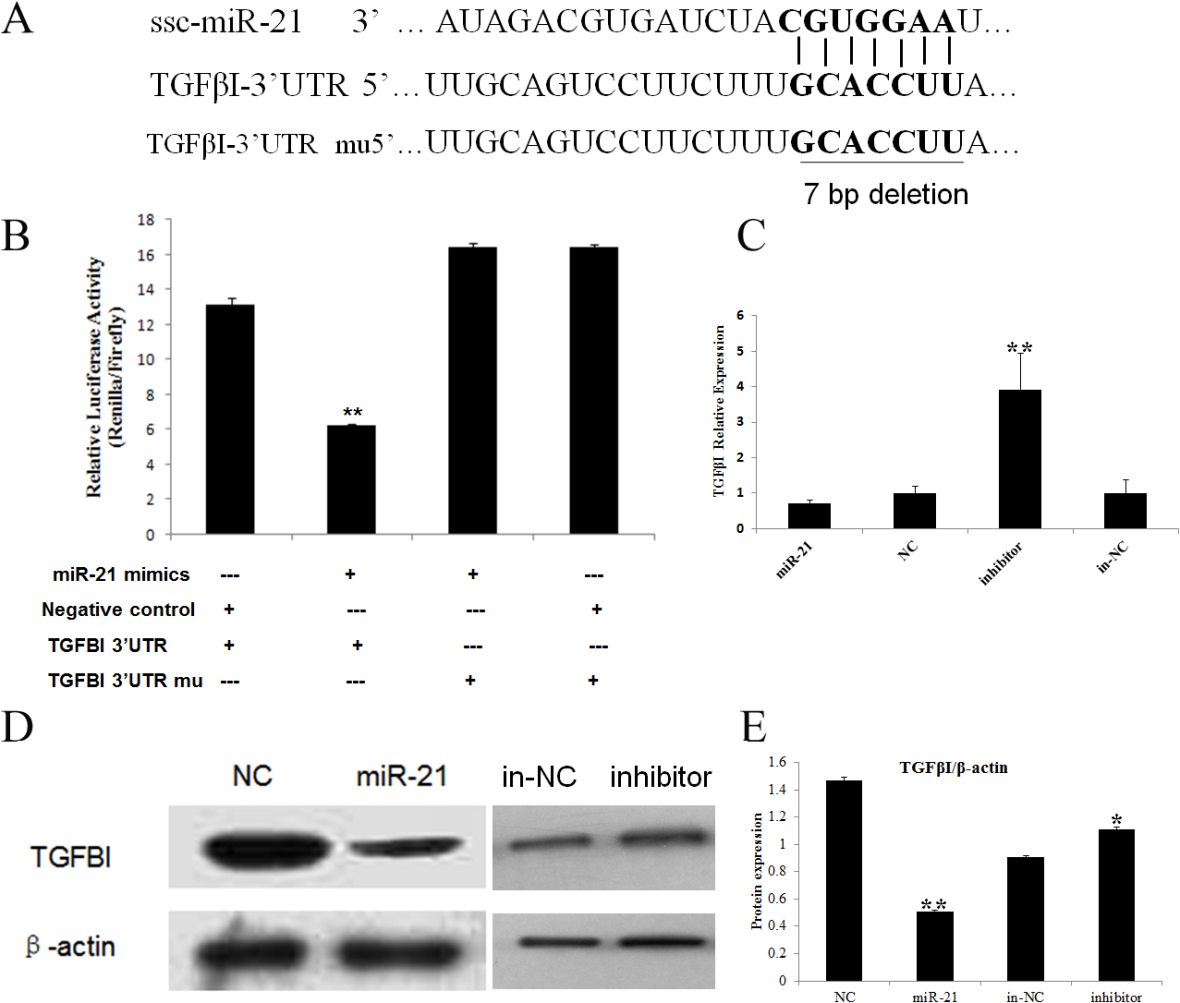
TGFβI is a target for miR-21. A. The predicted binding site between miR-21 and the TGFBI 3' UTR. B. Detection of luciferase activity in the PIEC cells after co-transfection with the luciferase reporter and miR-21/NC. The PIEC cells were co-transfected with the psi-check2-TGFβI 3’ UTR and miR-21 mimics/negative control duplex. The relative luciferase activity was measured after 48 h. The data represent the mean ± SD from three independent experiments performed in duplicate. C. RT-qPCR was performed to verify the decreased TGFβI expression following the overexpression of miR-21 and enhanced expression after miR-21 inhibition in the PIEC cells. The data represent the mean ± SD from three independent experiments performed in duplicate. D. Total cell extracts were harvested at 48 h after transfection, and the TGFβI protein was analyzed via immunoblotting. E. All quantitative results were normalized byβ-actin and were shown as mean ± SD from three independent experiments.

To understand the biological function and regulatory mechanism of ssc-miR-21 in skeletal muscle development, TargetScan (version 6.2) and miRanada were used to predict the putative targets of miR-21. A total of 164 potential targets were identified, and we used the DAVID gene annotation tool (http://david.abcc.ncifcrf.gov/) to further delineate the possible roles and mechanisms of ssc-miR-21 and its targets in mediating muscle development. Finally, we focused on the TGFβI gene in our study (Fig 3A). TGFβI can regulate the growth and differentiation of various cells. In myogenesis, its spatial and temporal expression has been correlated with the fibrous compositions of the surrounding myotubes [34]. Cusella-De Angelis et al. has reported that TGFβI inhibits the differentiation of fetal myoblasts but does not have negatively effect on embryonic myoblasts [35]. In mature adult muscle, TGFβI inhibits skeletal muscle regeneration by repressing satellite cell proliferation, myofibers fusion, and the expression of a set of muscle-specific genes [36]. Furthermore, TGFβI induces the transformation of myogenic cells into fibrotic cells after injury [37]. Here, we postulate that miR-21 may have played a regulatory role in skeletal muscle myogenesis through the suppression of TGFβI expression, thereby governing cell growth, survival and cellular homeostasis.

### Validating TGFβI as a regulatory target for miRNA-21

To validate whether TGFβI is directly targeted by ssc-miR-21 in the pig, we engineered a luciferase reporter that included either the wild-type or the mutant 3’ UTR of TGFβI, and a dual luciferase assay was performed using the PIEC cells. The co-expression of the luciferase reporter and the ssc-miR-21-mimics/negative control (NC) revealed that the luciferase activity of the wild-type 3’ UTR, which contained the ssc-miR-21 binding sites, was significantly decreased compared to that of the negative control. Moreover, the mutations of the putative binding sites at the TGFβI 3’ UTR abrogated this repression, supporting the direct interaction of ssc-miR-21 with the TGFβI 3’ UTR (Fig 3B).

We further examined the effects of ssc-miR-21 on endogenous TGFβI at both the mRNA and protein levels in the PIEC cells. The over-expression of ssc-miR-21 led to decreased TGFβI mRNA levels by 29.21% compared to the negative control, and miR-21 inhibitor could exert potent effects on TGFβI expression (Fig 3C). Immunoblotting results showed that over-expression of miR-21 attenuated TGFβI protein, whereas miR-21 inhibitor significantly promoted TGFβI protein in PIEC cells. (Fig 3D and 3E), All of these confirmed that miRNAs function as post-transcriptional repressors act by suppressing their target genes.

### Expression analysis of ssc-miRNA-21 and TGFβI during skeletal muscle development

**Fig 4:**
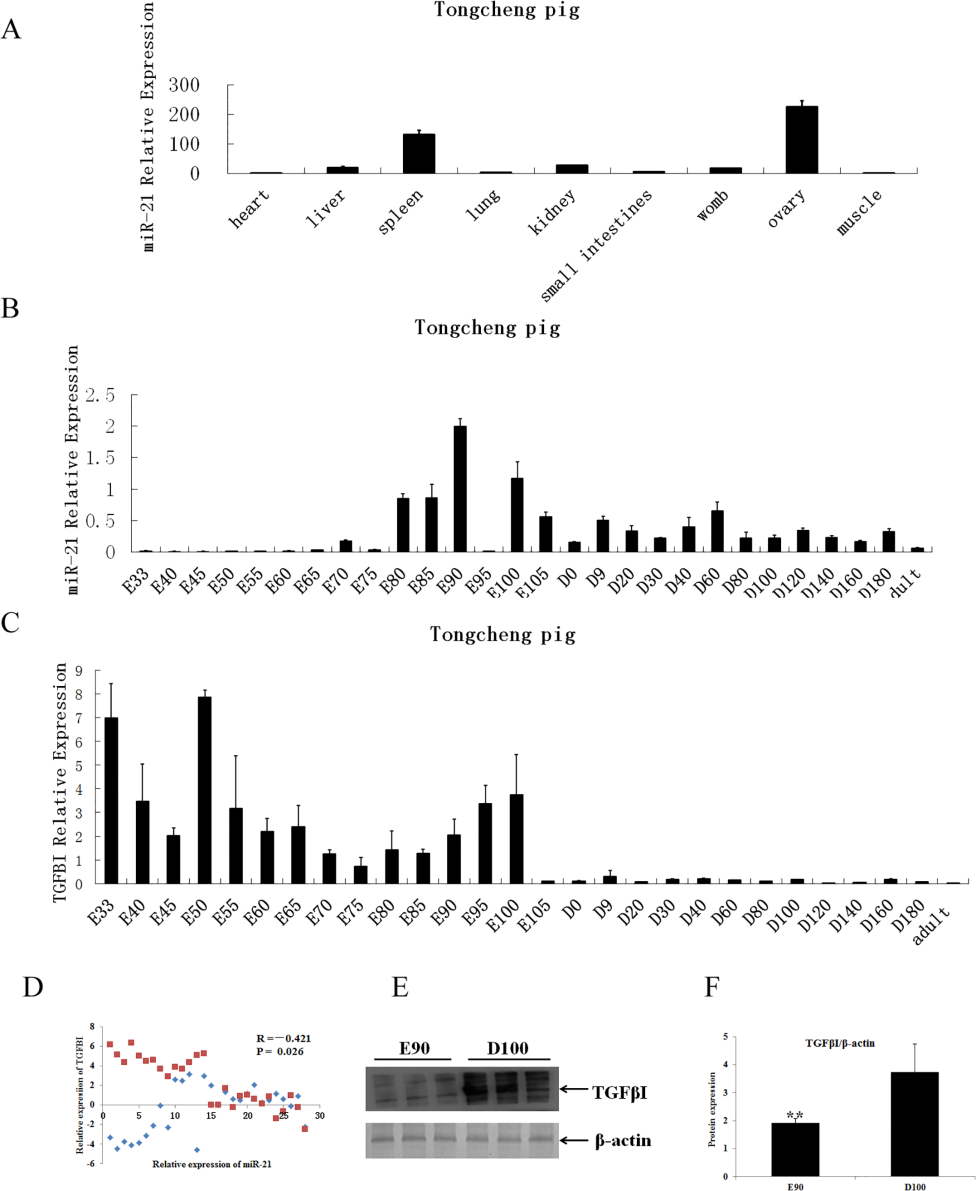
Expression profiles of miR-21 and TGFβI in Tongcheng pigs. A. Tissue distribution of miR-21. Total RNAs were extracted from the heart, liver, spleen, lung, kidney, small intestine, uterus, ovary and longissimus dorsi tissues. B. Expression profiles of miR-21 at different developmental stages. Total RNAs were extracted from the longissimus dorsi of the Tongcheng pigs at different dpcs (E) and postnatal days (D). The relative expression of miR-21 was compared to the internal control reference U6 and was analyzed using ΔΔCt. The data represent the mean ± SD from four independent experiments performed in duplicate. C. Expression profiles of TGFβI at different developmental stages. Analyses were performed according to B. D. Correlation between miR-21 and TGFβI expression during myogenesis in pigs. E. TGFβI protein levels were analyzed in extracts from the longissimus dorsi on E90 and D100 via immunoblotting. F. All quantitative results were normalized byβ-actin and were shown as mean ± SD from three independent experiments.

We subsequently analyzed the expression of ssc-miR-21 in nine different tissues (heart, liver, spleen, lung, kidney, small intestine, uterus, ovary and longissimus dorsi) of the adult Tongcheng pigs. The results showed that ssc-miR-21 expression varied greatly among the various tissues; it was expressed highly in the ovaries, moderately in the spleen, and only slightly in the other tissues (Fig 4A). Postnatally, ssc-miR-21 was expressed at low levels in the myocardial and longissimus dorsi tissues.

To further explore the functions of ssc-miR-21 in skeletal muscle development, we measured its dynamic expression in the skeletal muscle at 28 developmental stages (33, 40, 45, 50, 55, 60, 65, 70, 75, 80, 85, 90, 95, 100 and 105 dpc and 0, 9, 20, 30, 40, 60, 80, 100, 120, 140, 160 and 180 dpn and in adult pigs) using quantitative PCR. As shown in Fig 4B, ssc-miR-21 exhibited dynamic expression in the prenatal and post-natal skeletal muscles. In the prenatal skeletal muscle, ssc-miR-21 expression was maintained at generally low levels from 33 to 80 days. From 80 to 105 days, the levels first sharply increased then peaked at 90 days and subsequently decreased and were then maintained at stable low levels during the postnatal stage. These findings indicate that miRNA-21 may play an important role during prenatal myogenesis.

Next, we assessed the expression of TGFβI in the skeletal muscle at the 28 stages in which ssc-miR-21 was detected. Our results showed that the expression of TGFβI was high at 33 dpc, decreased from 40–45 dpc, peaked at 50 dpc, decreased from 55–85 dpc, and increased from 90–100 dpc after which they sharply decreased to nearly undetectable levels. Overall, the expression of TGFβI was greater in the prenatal skeletal muscle than in both the neonatal and adult muscles (Fig 4C). These observations suggest that the TGFβI gene likely played an important role in the development of prenatal skeletal muscle.

Considering the inverse correlation observed between the miRNA and target gene expression, we assayed the co-expression and abundances of ssc-miR-21 and TGFβI in the skeletal muscle. The results showed that the expression levels of ssc-miR-21 and TGFβI were significantly negatively correlated (r = -0.421, P<0.05) during the 28 stages evaluated in the Tongcheng pigs (Fig 4D).

Lastly, we extracted E90 and D100 longissimus dorsi proteins to confirm the TGFβI expression levels via western blotting. The results showed that TGFβI protein levels were up-regulated at D100 (Fig 4E and 4F). Based on these in vivo and in vitro experiments, we concluded that ssc-miR-21 directly regulates endogenous TGFβI protein synthesis during myogenesis.

### ssc-miRNA-21 plays a positive role by promoting PI3K/Akt/mTOR signaling

**Fig 5:**
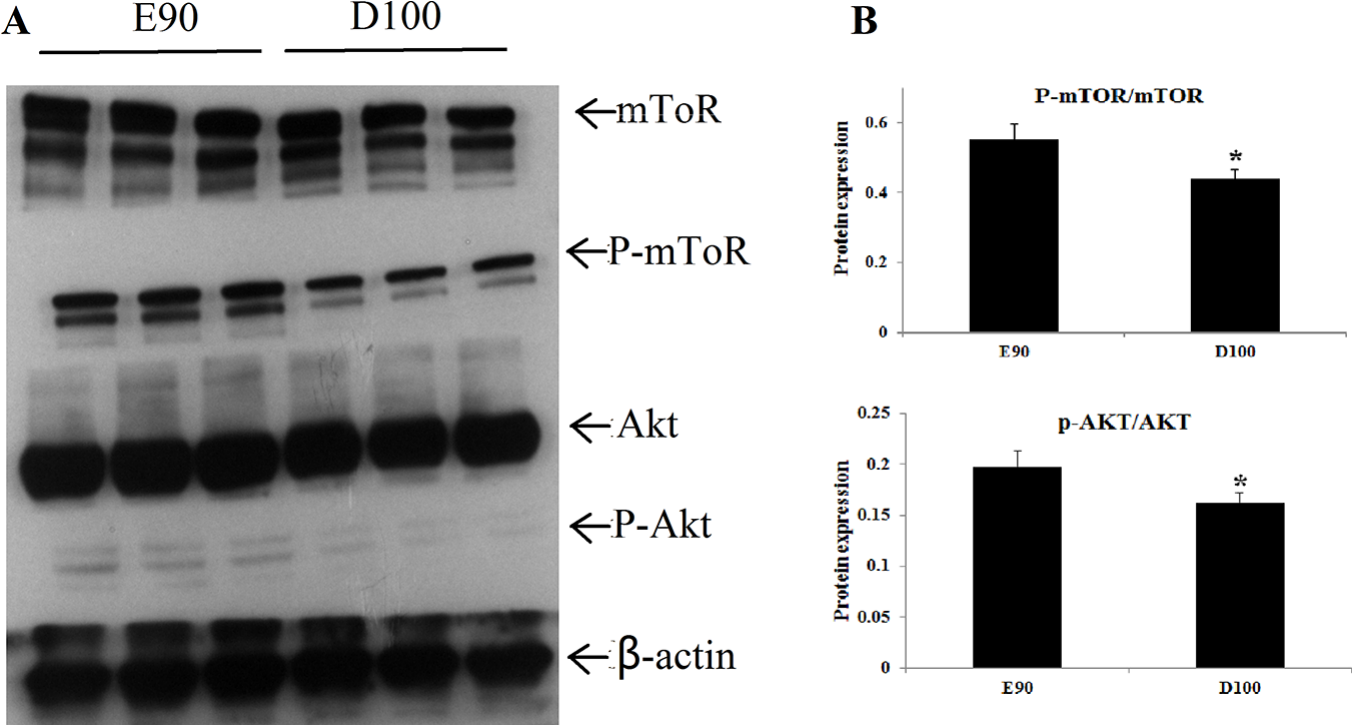
The potentiation of the Akt/mTOR pathway at E90 and D100. **A.** To detect the activation of the PI3K/Akt/mTOR signaling pathway, E90 and D100 longissimus dorsi were prepared and resolved by 10% SDS-PAGE. The PVDF membrane was probed with antibodies to phosphor-Akt and phosphor-mTOR, respectively. Antibodies against Akt, mTOR and β-actin were used to verify the equal protein loading. B. All quantitative results were normalized byβ-actin and were shown as mean ± SD from three independent experiments.

Increasing evidence has demonstrated that the PI3K/Akt cascade is critical to cell proliferation and protein synthesis. Therefore, we explored the associations of ssc-miR-21 and TGFβI expression with alterations in the *PI3K/Akt/mTOR* pathway. First, we assessed the phosphorylation statuses of Akt on E90 and D100. As shown in Fig 5A and 5B, expression was much stronger at E90 compared to D100, although total Akt protein levels were equal in both samples. The mTOR gene is a well-known downstream target of the PI3K/Akt pathway. Hence, we next examined the phosphorylation status of Ser2448 in mTOR, which is an indicator of its activation. The western blot indicated that the p-mTOR protein significantly increased on E90 compared with D100. These results suggest that miR-21 affected PI3K/AKT/mTOR signaling by suppressing TGFβI during swine skeletal muscle development.

## Discussion

Deep sequencing is a powerful tool for the profiling of known miRNAs and the discovery of novel miRNAs that have not been detected using traditional methods. Furthermore, it is a more sensitive and reliable technique for measuring miRNA levels compared with microarrays [38]. Our Solexa sequencing analysis detected a number of miRNAs that have been reported previously to be expressed in mammalian muscle (S3 Table). The abundance of ssc-miR-206 is consistent with its well-established activities during skeletal muscle development and its reported role in inducing differentiation in mouse C2C12 cells and during chicken myogenesis [4, 38, 39]. Changes in miRNA levels during fetal development were accompanied by endogenous changes in the levels of their known mRNA targets and inducers. We found that miR-1 was expressed at higher levels on D100 and that miR-378 was expressed at higher levels on E90. These data were consistent with the previously described role of miR-1 in regulating skeletal muscle satellite cell proliferation and differentiation by repressing Pax7 and that of miR-378 in targeting the myogenic repressor MyoR during myoblast differentiation [26,40].

In addition to alterations in muscle-specific miRNA levels, changes in the abundance of ubiquitously expressed miRNAs were also observed. Ssc-let-7a, ssc-let-7f, ssc-let-7c were highly expressed throughout prenatal and postnatal development in our study. The ssc-let-7 family is a conserved family of miRNAs. miR-23a inhibits myogenic differentiation by down-regulating the expression of fast myosin heavy-chain isoforms [41]. miR-26a targets the histone methyltransferase enhancer of Zeste homolog 2 during myogenesis [42]. miR-29 negatively regulates skeletal myogenesis by targeting Ring1 and YY1-binding protein (Rybp) [43]. miR-100 regulates neovascularization by suppressing rapamycin in endothelial and vascular smooth muscle cells [44]. All of these miRNAs could be found in our database, suggesting that our Solexa sequencing results were reliable. Compared to the Solexa deep-sequencing results, we detected 229 and 209 known miRNAs in the skeletal muscle on E90 and D100, respectively, suggesting that more miRNAs participated in prenatal myogenesis, during which more complex expression and regulatory patterns may have been present.

Mammalian skeletal muscle is not a homogeneous tissue [45]. During fetal myogenesis, primary fibers form first. Then, secondary fibers are generated from the myoblasts around the primary fibers [17]. Researchers have determined the precise spatiotemporal aspects of myogenic regulation. Wigmore et al. have revealed that porcine primary fibers mainly form at 38 to 64 dpc and secondary fibers at 54 to 90 dpc [46]. Tang et al. found that gene expression patterns differ significantly in Tongcheng and Landrace pigs at approximately 35 dpc, 60 dpc, 90 dpc [47]. In the present study, the fetal time point E90 was selected to coincide with important events in muscle development, particularly those occurring at the culmination of secondary fiber formation. We found that miR-21 was present at the highest levels at 90 dpc, after which the levels decreased throughout development and into adulthood. Considering that high levels of miR-21 were observed at the last key myogenesis time point, we hypothesize that miR-21 may be expressed in a tissue-specific and/or stage-specific manner to promote myoblast growth and ultimately promote muscle fiber formation during the prenatal stages in Tongcheng pigs.

The miR-21 participates in the formation of multiple tissues, including the secretory gland, lungs and kidney [48]. The overexpression of miR-21 may remarkably enhance the proliferation and migration of the vascular smooth muscle cells of the lungs [49]. Chen et al. reported that during the development of vertebrate models (such as zebrafish), miR-21 can be observed after 12 hours in the embryo and accounts for 40% of all miRNAs [50]. In our study, we found that miR-21 was expressed throughout the entire period of skeletal muscle development in the Tongcheng pigs. The levels of miR-21 were lower at 33 days during the embryonic stage and higher during the later embryonic stages, thus, it may be inferred that miR-21 played positive roles in the formation and growth of muscle fibers (leading to increased lengths and diameters). Because the target prediction analysis of this study indicated a potential pairing between miR-21 and the 3’ UTR region of TGFβI and considering the biological function of TGFβI in the process of myogenesis, we concluded that TGFβI was a candidate target gene for miR-21. Dual-luciferase reporter analysis demonstrated that miR-21 directly targeted the 3’ UTR of TGFβI, and mutations in the binding site abolished the suppression of luciferase activity by miR-21. To evaluate the myogenic roles of miR-21 and TGFβI, we examined the dynamic expression patterns in the skeletal muscle at 28 developmental stages. The results revealed that miRNA-21 and TGFβI were negatively correlated (r = -0.421, P = 0.026) at the mRNA level during the 28 stages of skeletal muscle development. The TGFβI protein was more abundant on D100 than on E90. These results suggest that miR-21 regulated skeletal muscle development by targeting TGFβI.

**Fig 6:**
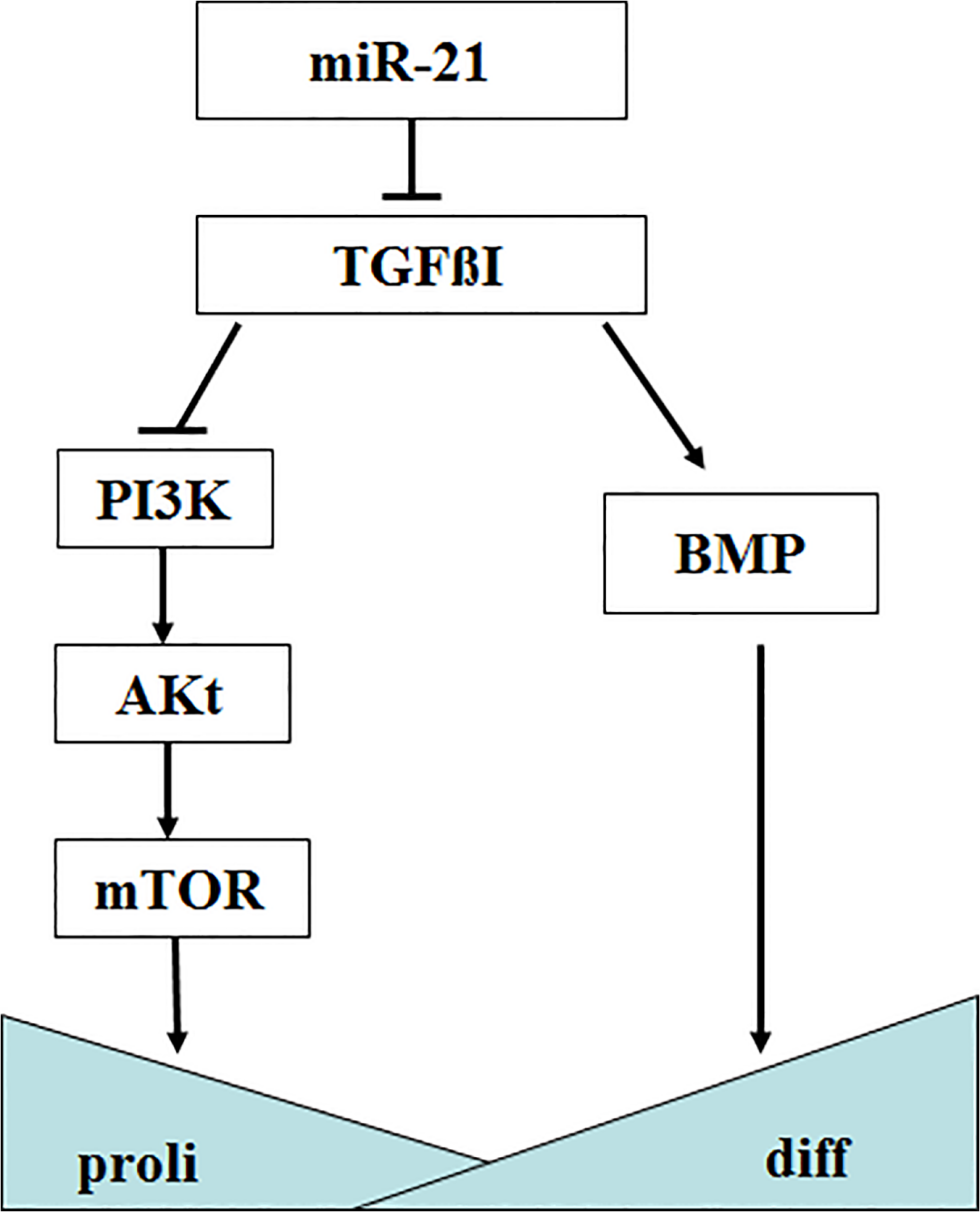
Crosstalk between miR-21, the target gene TGFβI and the PI3K/Akt/mTOR signaling pathway in myogenesis.

The mechanism underlying the role of TGFβI in mediating the development of skeletal muscle has not been elucidated to date. Wen et al. demonstrated that in the absence of TGFβI, mesothelial and mesothelioma cell lines exhibit elevated proliferation rates, enhanced plating efficiencies, increased anchorage-independent growth, and increased cellular protein synthesis rates via the suppression of the PI3K/Akt/mTOR signaling pathway [12]. Li et al. revealed that TGF-β up-regulates the expression of TGFβI in the TGF-β/BMP signaling pathway, which further induces the differentiation of bone marrow stem cells [51]. In the present study, we hypothesized that miR-21 facilitated myogenesis by regulating the PI3K/Akt/mTOR pathway (Fig 6). This pathway is well-known for its crucial role in cell growth and survival. We postulate that at E90, in the absence of TGFβI, PI3K induced the production of phosphatidylinositol-3, 4, 5-triphosphates (Ptdlns(3,4,5)P3), after which the Akt cascade was activated. Akt is a major mediator in the regulation of diverse cellular processes through its downstream effects on mTOR, thus resulting in a more activated PI3K/Akt/mTOR signaling pathway and associated biological functions.

Taken together, we identified miRNA-21 as a new myogenesis miRNA and documented that miR-21 directly affected PI3K/Akt/mTOR signaling by targeting TGFβI during skeletal muscle development in pigs. Our findings contribute to current knowledge of the roles of miRNA-21 and the molecular mechanisms of skeletal muscle development. In addition, this study has revealed candidate genes for the breeding of animals with improved traits for meat production.

## Supporting Information

S1 Fig Differentially expressed miRNAs in skeletal muscle at E90 and D100. (DOC)

S1 Table Primers for miRNA detection by RT-PCR and RT-qPCR. (XLS)

S2 Table Primers for miRNA-target identification. (DOC)

S3 Table Reads number and the distribution of small RNAs. (DOC)
