# Supplementary figures and images for: MicroRNA-21 Regulates PI3K/Akt/mTOR Signaling by Targeting TGFβI during Skeletal Muscle Development in Pigs
Source: PLoS One. 2015 May 7;10(5):e0119396. doi: 10.1371/journal.pone.0119396 (PMC4423774; doi:10.1371/journal.pone.0119396)

**
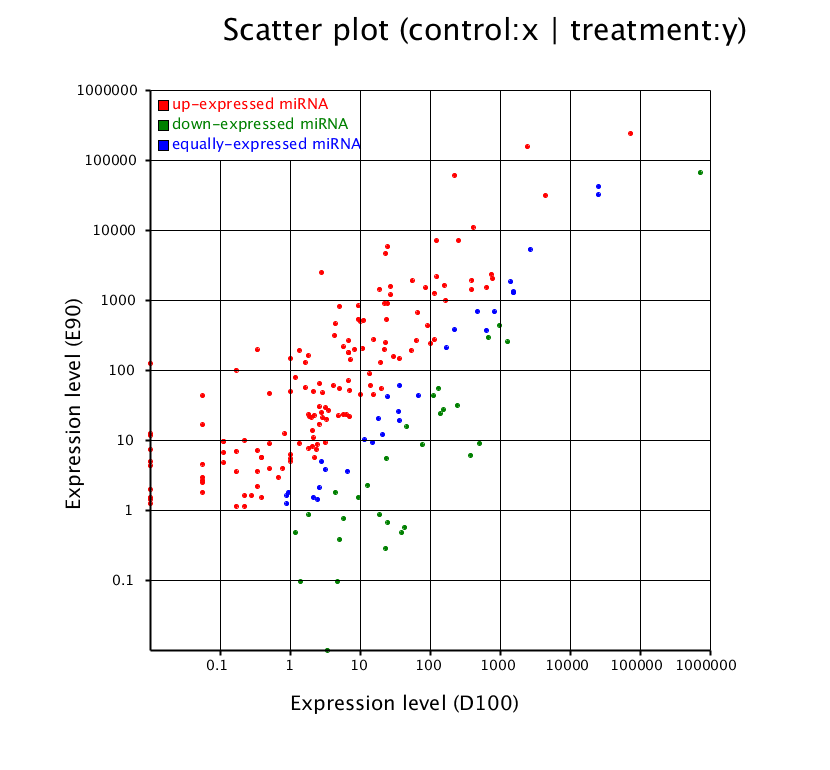
**

**Supplementary Fig. S1 Differentially expressed miRNAs in skeletal muscle at E90 and D100 (DOC)**

Supplement: S1 Fig — (DOC) [file pone.0119396.s001.doc]
