# Supplementary material for: MicroRNA-21 Regulates PI3K/Akt/mTOR Signaling by Targeting TGFβI during Skeletal Muscle Development in Pigs
Source: PLoS One. 2015 May 7;10(5):e0119396. doi: 10.1371/journal.pone.0119396 (PMC4423774; doi:10.1371/journal.pone.0119396)
Supplement: S2 Table — (DOC) [file pone.0119396.s003.doc]

**Table S2 Primers for luciferase reporter construction and RT-qPCR**

| Gene | Primer | Sequence（5'→3') | Product（bp） |
| --- | --- | --- | --- |
| TGFBI 3'UTR | Sense | CTCGAGCTTCCAGAGAGGACCTATCCCAAAT | 313 |
|  | Antisense | GCGGCCGCACACACCATGGCTCTGTCACAATAG |  |
| TGFBI (RT-  qPCR) | Sense | CTAAAGCCCACGAAACCTGA | 167 |
|  | Antisense | CACGGAAGAGTCCAAGCCAC |  |
| GAPDH | Sense | ATGGTGAAGGTCGGAGTGAAC | 235 |
|  | Antisense | CTCGCTCCTGGAAGATGGT |  |
