# Supplementary material for: MicroRNA-21 Regulates PI3K/Akt/mTOR Signaling by Targeting TGFβI during Skeletal Muscle Development in Pigs
Source: PLoS One. 2015 May 7;10(5):e0119396. doi: 10.1371/journal.pone.0119396 (PMC4423774; doi:10.1371/journal.pone.0119396)
Supplement: S3 Table — (DOC) [file pone.0119396.s004.doc]

**Table S3 Primers for miRNA RT-qPCR**

| miRNA ID | primer | sequence (5'→3') |
| --- | --- | --- |
|  |  |  |
| ssc-let-7a | SP | GGGTGAGGTAGTAGGTT |
|  | RT loop | CTCAACTGGTGTCGTGGAGTCGGCAATTCAGTTGAGAACTATAC |
| ssc-miR-10b | SP | GGGTACCCTGTAGAACC |
|  | RT loop | CTCAACTGGTGTCGTGGAGTCGGCAATTCAGTTGAGACAAATTC |
| ssc-miR-21 | SP | GTGCAGGGTCCGAGGT |
|  | RT loop | GTCGTATCCAGTGCAGGGTCCGAGGTATTCGCACTGGATACGACTCAACA |
| ssc-miR-30d | SP | GGGTGTAAACATCCCCGA |
|  | RT loop | CTCAACTGGTGTCGTGGAGTCGGCAATTCAGTTGAGGCTTCCAG |
| ssc-miR-127 | SP | GGGTCGGATCCGTCTG |
|  | RT loop | CTCAACTGGTGTCGTGGAGTCGGCAATTCAGTTGAGGCCAAGCT |
| ssc-miR-148a | SP | GGGTCAGTGCACTACAG |
|  | RT loop | CTCAACTGGTGTCGTGGAGTCGGCAATTCAGTTGAGACAAAGTT |
| ssc-miR-181a | SP | GGGAACATTCAACGCTGT |
|  | RT loop | CTCAACTGGTGTCGTGGAGTCGGCAATTCAGTTGAGACTCACCG |
| ssc-miR-199*-3P | SP | GGGACAGTAGTCTGCA |
|  | RT loop | CTCAACTGGTGTCGTGGAGTCGGCAATTCAGTTGAGAACCAATG |
| ssc-miR-378 | SP | GGGACTGGACTTGGAGT |
|  | RT loop | CTCAACTGGTGTCGTGGAGTCGGCAATTCAGTTGAGGCCTTCTG |
|  | universal AP | CTCAACTGGTGTCGTGGAGTC |
| U6 | SP | GCTTCGGCAGCACATATACTAAAAT |
|  | AP | CGCTTCACGAATTTGCGTGTCAT |

**Notes:** SP: sense primer, RT loop: primer for miRNA reverse transcript, AP: anti-sense primer, Universal AP: universal anti-sense primer for miRNA qPCR.
